# Changes in immunological parameters by ageing in rural healthy Indian adults and their associations with sex and lifestyle

**DOI:** 10.1038/s41598-022-19227-z

**Published:** 2022-09-02

**Authors:** Dhiraj Agarwal, Sourav Paul, Pallavi Lele, Vikrant Piprode, Anand Kawade, Neerja Hajela, Ashish Bavdekar, Varsha Parulekar, Manisha Ginde, Gandhali Paranjape, Kazunori Matsuda, Tetsuji Hori, Sanjay Juvekar, Girdhari Lal

**Affiliations:** 1 KEM Hospital Research Centre, Vadu Rural Health Program, Pune, Maharashtra 412216 India; 2 National Centre for Cell Science, NCCS Complex, Ganeshkhind, Pune, Maharashtra 411007 India; 3 Paediatrics Department, KEM Hospital Research Centre, Pune, Maharashtra 411011 India; 4 Yakult Danone India Pvt. Ltd., 212, Ground Floor, Okhla Industrial Estate Phase-III, New Delhi, Delhi 110020 India; 5 DiagnoSearch Life Sciences Pvt. Ltd., 702, Dosti Pinnacle Plot No. E-7, Road No. 22 Wagle Industrial Estate, Thane, Maharashtra 400604 India; 6 Yakult Central Institute, 5-11 Izumi, Kunitachi-Shi, Tokyo, 186-8650 Japan

**Keywords:** Immunology, Adaptive immunity, Antimicrobial responses, Cytokines, Innate immune cells, Innate immunity, Lymphocytes, Vaccines

## Abstract

Several factors including sex and lifestyle have been reported to contribute to the age-related alteration of immune functions. The study was undertaken to determine age-related differences in the proportion of peripheral blood mononuclear lymphocytes in the Indian population using blood samples from 67 healthy adults (33 females and 34 males) aged between 20 and 80 years old. In the linear regression analysis to estimate the relationship with age categories, there was a significant increase in the frequency of natural killer cells with ageing, while their cytolytic activity significantly declined. The frequency of CD4^+^ T cells increased with age, whereas that of CD8^+^ T cells decreased, resulting in the age-associated increase of the CD4/CD8 ratio. The subsets of B cells did not show any significant relationship with age. Although there were variations between the male and female subgroups in effect size of ageing, the trends were in the same direction in all the parameters. Reduced fat intake was associated with a lower frequency of CD4^+^ T cells, and higher serum cotinine level was associated with a higher CD4/CD8 ratio. The results indicate that cellular immunity in the Indian population is affected by ageing, while humoral immunity is less susceptible to ageing.

## Introduction

Humans are regularly exposed to a plethora of infectious agents like bacteria, viruses, fungi, and parasites, resulting in an infection that can be asymptomatic or that can lead to a spectrum of diseases. The cellular and humoral immune responses play a critical role in detecting and clearing pathogens^1^. The intensity of the immune response is highly variable between individuals and is influenced by several genetic and non-genetic factors, including age, gender, metabolic, and nutritional factors^1^. Among these, age is one of the most well-known factors, and a decline in immune function with age is known as ‘immunosenescence’ which is characterized by an impairment of different immunological parameters, including adaptive and innate immune cells^2,3^.

Low natural killer (NK) cell activity levels have been observed in the elderly and those with autoimmune diseases and cancer^4–6^. In contrast, the elderly, where the NK cells are well preserved both in numbers and function, help them lead healthier and longer lives^7^. Animal and some human studies have shown an alteration in cell numbers and activity of T cell subpopulations (CD4^+^ and CD8^+^ T cells) with age. CD8^+^ T cells, Th1 CD4^+^ T cells, NK cells, and NK T cells produce inflammatory cytokine interferon gamma (IFN-γ) ^8^. IFN-γ is a cytokine that promotes innate and adaptive immune responses to microbial infection and spontaneous and therapeutic anti-tumour immunity^9–11^. IFN-γ promotes differentiation of Th1 cells and NK cell cytotoxic activity, increasing the antigen processing and major histocompatibility complex (MHC) molecule expression on antigen-presenting cells^8,12,13^. The level of IFN-γ generally tends to decrease with age^14^. The decline in both qualitative (various types of immune cells) and quantitative levels (frequency/number of specific immune cells) of immune response with ageing results in increased susceptibility to infection, compromises the ability to fight infection and increases mortality, malignancies, and autoimmune diseases^15^.

Besides age, sex-specific differences in immune response have previously been observed for many bacterial and viral pathogens^16,17^. It has been shown that geographical variation can influence immune response, as observed in studies conducted in Austria, France, Spain, Italy, Japan, and India^18–22^. However, multiple immune parameters, including both acquired and innate immune cells in a rural locations in different age groups in Western India have not been well studied.

This study aimed to obtain the baseline information about immune parameters, including NK cells and subsets of T cells and B cells in healthy rural Indian adults aged between 20 and 80 years old, and determine age-associated changes in the composition. Further, we assessed their sex and lifestyle factors variations, including dietary patterns and nicotine intake. The study will provide key new findings that will help evaluate dietary interventions such as functional foods to improve immune functions in India.

## Materials and methods

### Study site and participants

The study was conducted in 22 villages in the Pune district covered by the Vadu Health and Demographic Surveillance System (HDSS) of KEM Hospital Research Centre (Pune, Maharashtra, India) from September 2017 to January 2018 as an observational study. The study area was primarily rural and included a few villages that had undergone semi-urbanization. Participants were randomly selected through stratification according to sex and age categories (21–30, 31–40, 41–50, 51–60, 61–70, and 71–80 years) to achieve a minimum of 60 eligible ones. To achieve the same, it was planned to collect samples from 120 individuals (with 100% oversampling) using Vadu HDSS (round 27) database as a sampling frame. The first stage of enrolment was designed such that 84 study participants were to have consented. The participants were excluded if they had any known/reported allergy or hypersensitivity to specific medicines and foods, acute or chronic illness, or had a high fever of 38 °C at blood sampling. Out of the total 84 participants approached for the study, 1 found to be dead, 3 out-migrated, 3 refused to participate, 10 screened failed, 67 participants were eligible for analysis, and the age-stratified number was as follows: 11 participants (5 females, 6 males) were in the age group of 21–30 years, 13 in 31–40 years (7 females, 6 males), 11 in 41–50 years (6 females, 5 males), 12 in 51–60 years (5 females, 7 males), 10 in 61–70 years (5 females, 5 males), and 10 in 71–80 years (5 females, 5 males). We evaluated lifestyle factors affecting immune parameters. The participants were asked to fill out a questionnaire to check whether participants consumed a healthy diet or not. We defined a healthy diet as the participant who eats at least five servings of fruit and/or vegetables each day; reduces intake of salt in the diet; reduces intake of fat in the diet; limits consumption of processed foods; avoids eating foods prepared outside of a home with response to each question as Yes or No.

### Blood collection

Standard phlebotomy protocol was followed for blood sample collection, ensuring the safety of study participants as well as the team member. Study participants were asked to fast for at least 8 to 10 h before blood collection. Venous blood (25–30 ml) was collected in heparin-containing sterile tubes (BD Biosciences; San Jose, CA, USA). The whole blood sample was immediately shipped to the National Centre for Cell Science, Pune, India, at 2–8 °C in an insulated box for processing and analysis.

### Isolation of PBMCs and NK cells

Peripheral Blood Mononuclear Cells (PBMCs) were isolated using Lymphoprep and SepMate PBMC isolation tubes (Stem Cell Technology; Vancouver, Canada) as per the manufacturer’s guidelines. NK cells from the blood were purified using the RosetteSep antibody cocktail (Stem Cell Technologies) as per the manufacturer’s guidelines. Purified NK cells were used for the assays testing NK cell activity.

### Analysis of NK cell activity

NK cell activity was measured in terms of the cytolytic function and degranulation potential. NK cell cytolytic activity on K562 target cells was measured by the CFSE-based assay (direct assay)^23^. The degranulation potential of NK cells after co-culturing with K562 target cells was measured by the CD107-based degranulation assay (indirect assay)^23,24^. In a comparison of the results between the two assays, there was a significant positive linear correlation (Fig. S1; ρ = 0.523, *p* < 0.001).

#### Direct assay

The human leukaemia K562 cell line was used as a target for NK cell lytic activity. K562 cell line was cultured in RPMI-1640 medium (HiMedia; Mumbai, India) supplemented with 10% FBS and antibiotics (Penicillin 100U/ml, Streptomycin 100 mg/ml) in humidified 5% CO_2_ incubator at 37 °C. K562 cells were washed with Phosphate-buffered saline (PBS) and stained with 5 μM CFSE (Thermo Fisher Scientific; Waltham, MA, USA). Freshly-isolated NK cells (5 × 10^4^ cells/well) as effector cells and CFSE-labelled K562 cells as target cells were co-incubated at an effector/target (E/T) ratio of 10:1 at 37 °C in U-bottomed 96 well plates. After 5 h of incubation at 37 °C, cells were harvested and stained with APC-conjugated anti-human CD56 antibody (clone NCAM; BioLegend; San Diago, CA, USA), eFluor 450-conjugated anti-human CD3ε (clone UCHT1; BioLegend) and 7AAD (Sigma Aldrich; St. Louis, MO, USA) for 30 min in the dark. Cells were washed, and apoptosis of CFSE-labelled K562 cells was analyzed using Flow cytometry (FACS Canto II; BD Biosciences). Increased frequency of 7-AAD^+^CFSE^+^ cells represents apoptosis of the cells. Data were analyzed after gating on CFSE^+^CD56^−^CD3ε^−^ cells using FlowJo software (Tree Star; Ashland, OR, USA).

#### Indirect assay

Freshly isolated NK cells were used as effector cells and K562 cells as target cells. Effector and target cells were co-incubated at E:T ratio of 10:1 for 4 h at 37 °C. PE-conjugated anti-human CD107a antibody (10 μg/ml; clone H4A3; BioLegend) was added in the culture. Phorbol-12-myristate-13-acetate (50 ng/ml; Sigma Aldrich) and ionomycin (250 ng/ml; Sigma Aldrich) were used as a positive control, whereas medium alone was used as unstimulated control. After stimulation, cells were stained with APC-conjugated anti-human CD56 antibody, Alexa Fluor 488-conjugated anti-human CD3ε antibody. Cells were washed with a cold medium, acquired using flow cytometry (FACS Canto II, BD Bioscience), and analyzed after gating of CD56^+^CD3ε^−^ cells using FlowJo software. Higher CD107^+^CD56^+^ cells indicate increased degranulating NK cells.

### Analysis of T cells and intracellular IFN-γ staining

PBMCs (1 × 10^6^ cells) were stimulated in 1 ml complete RPMI 1640 medium in the presence of a cell stimulation cocktail containing protein transport inhibitor cocktail (BioLegend) in 24 well plates. Plates were incubated in 5% CO_2_ incubator at 37 °C for 6 h. Cells were harvested, surface stained for Alexa Fluor 488 anti-human CD3ε, BV421 anti-human CD4 (clone OKT4; BioLegend), and PE/Cy7 anti-human CD8 (clone SK1; BioLegend) on ice for 30 min. After washing with PBS, cells were fixed and permeabilized using Fix/Perm Buffer (BioLegend) as per manufacturer’s guidelines and stained with PE anti-human IFN-γ (clone 4S.B3; BioLegend). Cells were acquired using FACS Canto II and analyzed using FlowJo software.

### Analysis of B cells

PBMCs (1 × 10^6^ cells) were surface stained with APC-conjugated anti-human CD138 (clone MI15; BioLegend), APC-Cy7-conjugated anti-human CD19 (clone HIB19; BioLegend) BV510-conjugated anti-human CD27 (clone O323, BioLegend), BV421-conjugated anti-human CD38 (clone HB-7; BioLegend), PE-conjugated anti-human IgG (clone M1310G05; BioLegend), and Dylight 488-conjugated anti-human IgA antibody (Abcam; Cambridge, UK). Cells were washed, fixed with 1% paraformaldehyde solution, and acquired using FACS Canto II. Total CD19^+^CD27^+^CD38^hi^CD138^+^ plasma cells, CD19^+^CD27^+^CD38^+^CD138^−^ plasmablast cells, CD19^+^IgA^+^ B cells, and CD19^+^IgG^+^ B cells were analyzed.

### Measurement of serum cotinine level

Serum cotinine level was measured using Cotinine ELISA Kit (cat. no. KA0930; Abnova; Taipei, Taiwan) according to the manufacturer’s guidelines.

### Statistical analysis

The statistical analysis was performed in R v. 3.4.1 software (R Foundation for Statistical Computing; Vienna, Austria). There was neither priori sample size calculation nor hypothesis testing for the study. All the study parameters, including immunological parameters, are presented as descriptive statistics (Table 1). It includes the mean, standard error of the mean, median, minimum, maximum, and 95% confidence interval (CI) for the parameters. The Wilcoxon rank-sum test was employed to assess statistical differences in the data between the sex subgroups using the exactRankTests package. Further stratification by different age groups was also performed for the parameters. The R package glm was used for generalized linear regression analysis. In assessing the age-associated changes, the linear regression analysis used the age groups (categories were converted to numbers from 1 to 6 in age order) as a sole explanatory variable (without adjustment by any other parameters); and each of the immunological parameters as a response variable. Based on the assumption that the inter-individual variation of the immune parameters would be greater than the intra-individual yearly variation, the categorized age groups by decade were employed as the explanatory variable to increase sensitivity for detecting their age-associated changes. The assumptions of normality and homoscedasticity in the generalized linear regression (Table S1) were tested by the Shapiro–Wilk test and the Goldfeld-Quandt test using R packages of RVAideMemoire and lmtest, respectively (Table S2). As the assumption of normality turned out not to be met in most of the cases, we additionaly conducted non-parametric linear regression analysis based on Theil-Sen and Siegel method using the R package mblm to assure that the results were similar in the both analyses (Table S3). In evaluations of the effect of lifestyle factors on the immune parameters, each of the food habits questionnaire data (answers were converted to numbers: No = 0, Yes = 1) and the serum cotinine levels (< 20 ng/ml were regarded as ‘low = 0’ and that of ≥ 20 ng/ml was as ‘high = 1’, respectively) were used as a sole explanatory variable (without adjustment by any other parameters). The fixed effect size of the explanatory variable, and its 95% CI and *p*-value: Pr( > |t|) were estimated in a total of 67 subjects and in sex subgroups. A post-hoc power analysis was performed on the results obtained in the generalized linear regression analysis using G*Power v. 3.1.9.7. There Cohen’s *f*^2^ was determined from a squared correlation coefficient between the age category and the immune parameters, and which was then used as effect size to calculate observed power (1-β error probability). All statistical tests were performed at the 0.050 level of significance. All *p*-values were rounded to three decimal places and are presented as “*p* < 0.001” if they were below 0.001 after rounding. Owing to the observational character of the study, no correction for multiplicity testing was applied.

**Table 1:** Immunological parameters of the study population.

Item	Statistics	Female (*n* = 33)	Male (*n* = 34)
CD3^−^CD56^+^ NK cells (%)	Mean ± SD	13.03 ± 7.20	13.83 ± 8.19
	95% CI	[10.48, 15.58]	[10.98, 16.69]
	Median (min, max)	11.3 (2.4, 32.9)	12.6 (0.5, 33.7)
CD3^−^CD56^+^ NK cells (counts/10^5^ PBMCs)	Mean ± SD	6820.2 ± 4687.9	6568.8 ± 4561.3
	95% CI	[5158.0, 8482.5]	[4977.3, 8160.3]
	Median (min, max)	5481 (467, 18,540)	5481 (480, 19,264)
CFSE^+^7AAD^+^ target cells (%)	Mean ± SD	7.38 ± 4.45	5.89 ± 3.16
	95% CI	[5.80, 8.96]	[4.77, 7.02]
	Median (min, max)	5.7 (2.0, 21.9)	5.1 (1.6, 16.6)
CD107a^+^CD56^+^ NK cells (%)	Mean ± SD	8.89 ± 5.48	9.66 ± 6.01
	95% CI	[6.94, 10.83]	[7.56, 11.76]
	Median (min, max)	6.9 (2.4, 27.1)	8.25 (1.1, 26.8)
CD3^+^CD4^+^ T cells (%)	Mean ± SD	51.95 ± 11.37	50.26 ± 9.47
	95% CI	[47.92, 55.98]	[46.95, 53.56]
	Median (min, max)	52.6 (27.4, 75.2)	50.35 (33.1, 71.9)
CD3^+^CD8^+^ T cells (%)	Mean ± SD	27.61 ± 9.46	28.98 ± 10.29
	95% CI	[24.26, 30.97]	[25.39, 32.57]
	Median (min, max)	26.9 (10.0, 51.8)	30.7 (8.6, 46.2)
CD4/CD8 ratio	Mean ± SD	2.28 ± 1.51	2.17 ± 1.50
	95% CI	[1.74, 2.82]	[1.65, 2.70]
	Median (min, max)	1.88 (0.59, 7.37)	1.70 (0.72, 8.12)
IFN-γ^+^CD4^+^ T cells (%)	Mean ± SD	13.27 ± 5.66	13.01 ± 5.64
	95% CI	[11.26, 15.28]	[11.04, 14.98]
	Median (min, max)	11.9 (5.1, 30.5)	12.4 (4.9, 28.7)
IFN-γ^+^CD8^+^ T cells (%)	Mean ± SD	41.38 ± 16.49	41.67 ± 15.97
	95% CI	[35.54, 47.23]	[36.10, 47.25]
	Median (min, max)	38.9 (11.0, 72.6)	42.9 (13.2, 76.5)
CD19^+^ B cells (%)	Mean ± SD	16.01 ± 5.72	13.68 ± 6.14
	95% CI	[13.98, 18.04]	[11.53, 15.82]
	Median (min, max)	15.3 (7.2, 33.6)	11.6 (2.4, 26.5)
CD38^+^CD138^+^ plasma cells (%)	Mean ± SD	59.24 ± 10.32	60.50 ± 10.40
	95% CI	[55.45, 63.03]	[56.75, 64.25]
	Median (min, max)	59.9 (42.3, 78.8)	60.0 (41, 82)
CD38^+^CD138^−^ plasmablast cells (%)	Mean ± SD	40.75 ± 10.32	39.49 ± 10.40
	95% CI	[36.96, 44.53]	[35.74, 43.24]
	Median (min, max)	40.1 (21.2, 57.7)	40.0 (18.0, 59.0)
IgG^+^CD19^+^ B cells (%)	Mean ± SD	7.17 ± 3.60	6.33 ± 3.15
	95% CI	[5.85, 8.49]	[5.19, 7.47]
	Median (min, max)	6.1 (2.0, 15.0)	5.8 (2.6, 17.2)
IgA^+^CD19^+^ B cells (%)	Mean ± SD	7.47 ± 4.55	6.97 ± 3.33
	95% CI	[5.80, 9.14]	[5.77, 8.18]
	Median (min, max)	6.5 (2.8, 24.8)	6.5 (1.7, 15.2)

### Institutional review board

The study was conducted according to ICH-GCP and Indian ethical guidelines, and approved by the Ethics Committees of the KEM Hospital Research Centre, Pune, Maharashtra, India (Reference No. 1720) and the National Centre for Cell Science, Pune, Maharashtra, India (Reference No. NCCS/IEC/2017-II/1).

### Informed consent

Written informed consent was obtained from all the study participants involved in the study.

## Results

### Age-associated changes in NK cells

**Figure 1:**
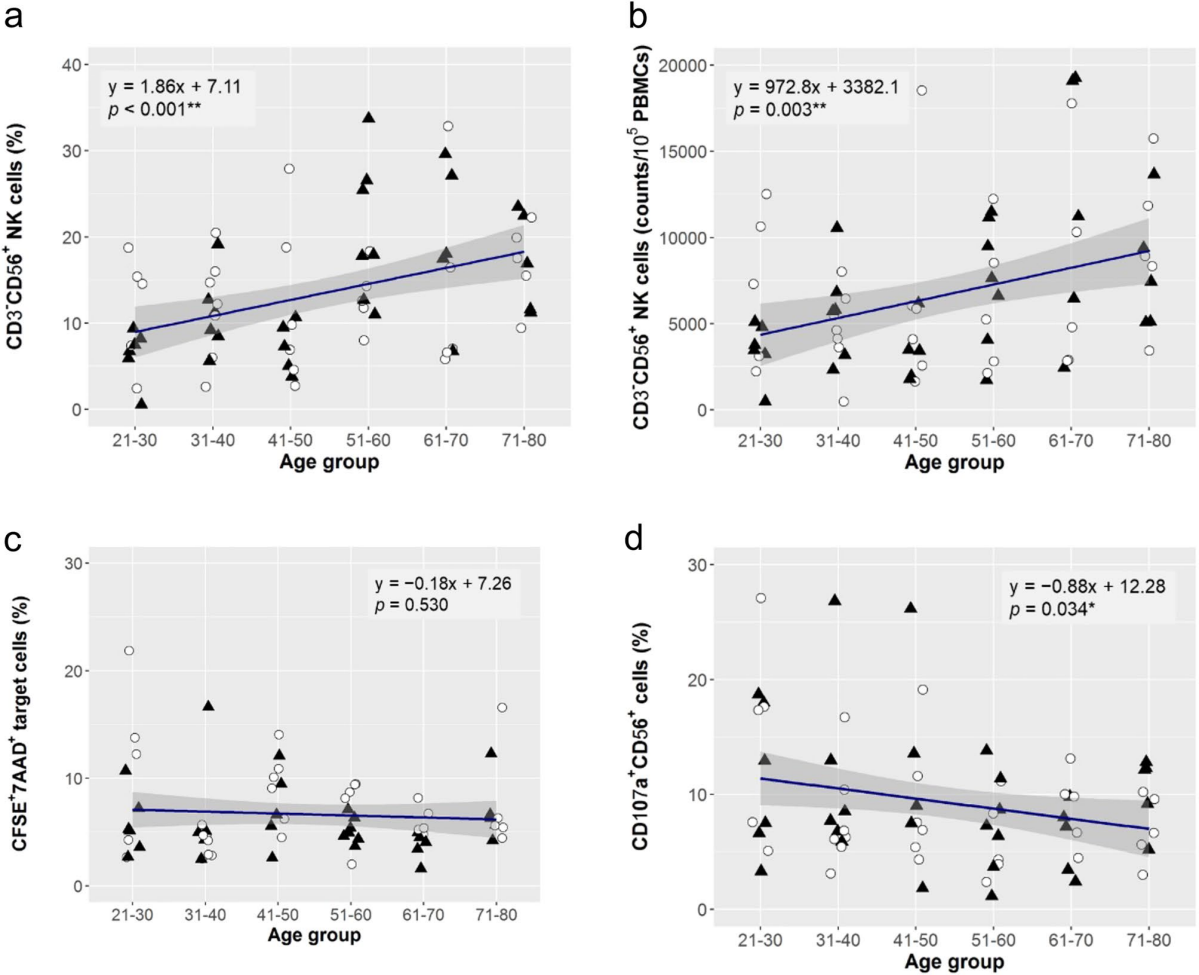
Age-associated changes in NK cells. Linear regression analysis to evaluate the effect of ageing on the percentage of CD3^−^CD56^+^ NK cells gated to total lymphocyte population (**a**), the number of CD3^−^CD56^+^ NK cells in 10^5^ PBMCs. (**b**), cytolytic activity of NK cells by direct assay (**c**), and degranulation potential of NK cells by indirect assay (**d**). The linear regression curve is plotted for the data of 67 subjects (33 females: open circle, 34 males: closed triangle), and its equation and *p*-value (*p < 0.050, **p < 0.010) for the effect size of ageing are shown. The statistics of the analysis are summarised in Table S1.

Among 70 participants initially enrolled in the study, 67 participants (33 females and 34 males) were eligible for the analysis. Blood sample was collected from each participant and analyzed for specific immune parameters. The descriptive statistics of the entire baseline data of the immune parameters are represented in Table 1. In a comparison between the sex subgroups, no statistical significance was observed in any of the immune parameters (*p* ≥ 0.050). We first analyzed the impact of ageing on NK cells by linear regression analysis. There were significant age-related increases of CD3^−^CD56^+^ NK cells both in percentage (estimate, 1.86; *p* < 0.001; observed power = 0.952; Fig. 1a) and absolute number in the peripheral blood mononuclear cells (PBMCs) (estimate, 972.8; *p* = 0.003; observed power = 0.871; Fig. 1b). As summarised in Table S1, these trends were stronger in the male subgroup (estimate 2.73 and 1279.4, respectively) than in the female subgroup (estimate 0.94 and 652.5, respectively). In the direct assay, there was no significant age-associated change in the NK cytolytic activity against K562 cells (Fig. 1c). In the indirect assay, it was observed that degranulation potential represented by the percentage of CD107a^+^CD56^+^ NK cells was significantly reduced with age (estimate, − 0.88; *p* = 0.034; observed power = 0.580; Fig. 1d), however its observed power was not strong enough to support for the observation and the trend diminished in both male and female subgroups (Table S1).

### Age-associated changes in T cell subsets

**Figure 2:**
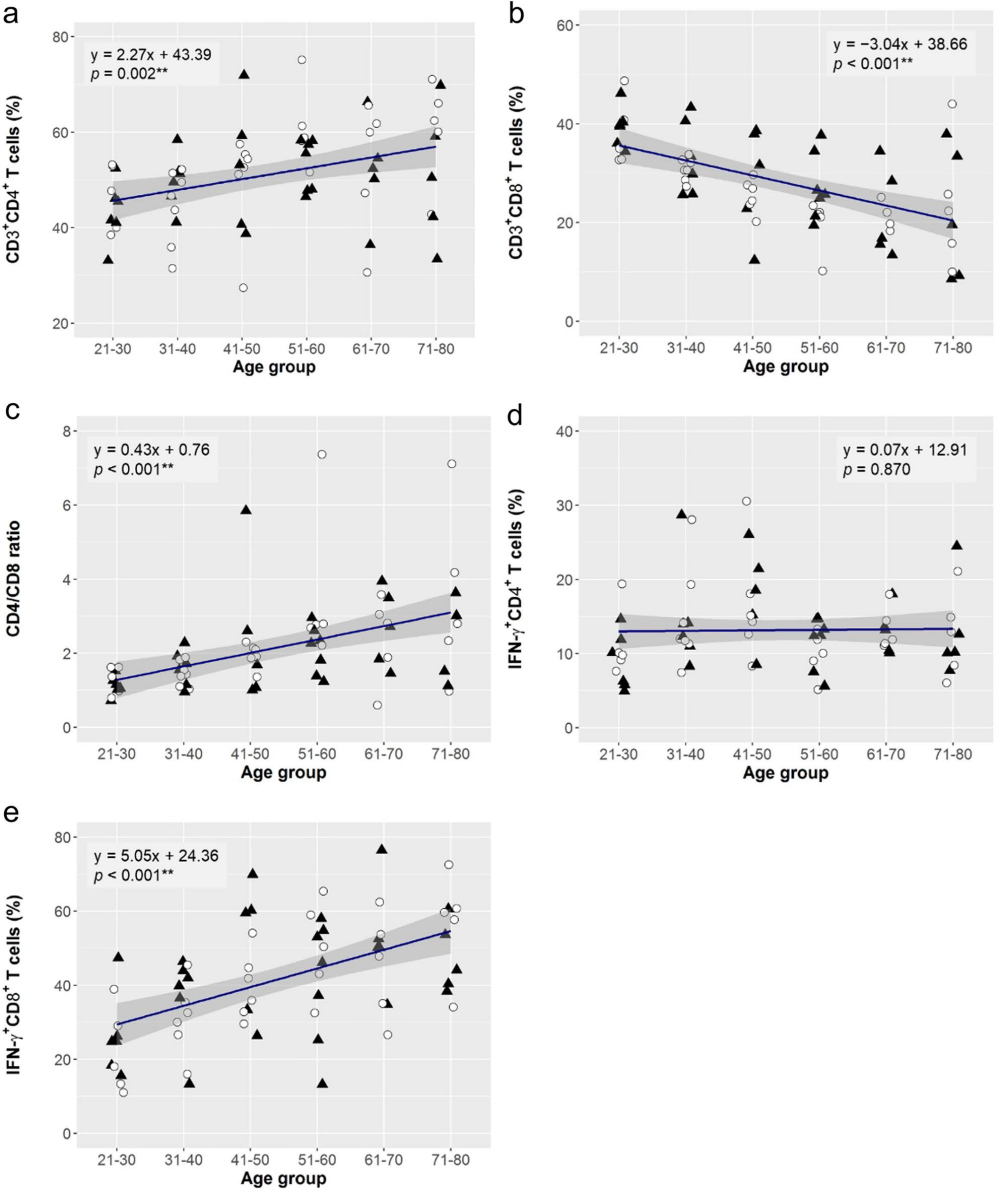
Age-associated changes in T cell subsets. Linear regression analysis to evaluate effect of aging on the frequencies of CD4^+^ T cells (**a**; CD3^+^CD4^+^ T cells), CD8^+^ T cells (**b**; CD3^+^CD8^+^ T cells), IFN-γ^+^CD4^+^ T cells (**d**) and IFN-γ^+^CD8^+^ T cells (**e**), and CD4/CD8 ratio (**c**). The linear regression curve is plotted for the data of 67 subjects (33 females: open circle, 34 males: closed triangle), and its equation and *p*-value (**p < 0.010) for the effect size of ageing are shown. The statistics of the analysis are summarised in Table S1.

The linear regression analysis showed a significant increase in the percentage of CD3^+^CD4^+^ T cells with age (estimate, 2.27; *p* = 0.002; observed power = 0.890; Fig. 2a), while that of CD3^+^CD8^+^ T cells significantly declined (estimate, − 3.04; *p* < 0.001; observed power = 0.999; Fig. 2b), resulting in an increase in CD4/CD8 ratio (estimate, 0.43; *p* < 0.001; observed power = 0.995; Fig. 2c). This trend was common in male and female subgroups (Table S1). IFN-γ is a mediator of the cell-mediated immune response of CD4^+^ T and CD8^+^ T cells. The mean percentage of IFN-γ^+^CD4^+^ T cells was stable with age (Fig. 2d), while that of IFN-γ^+^CD8^+^ T cells significantly increased with age (estimate, 5.05; *p* < 0.001; observed power = 0.999; Fig. 2e), which was common in both male and female subgroups (Table S1).

### Age-associated changes in B cell subsets

**Figure 3:**
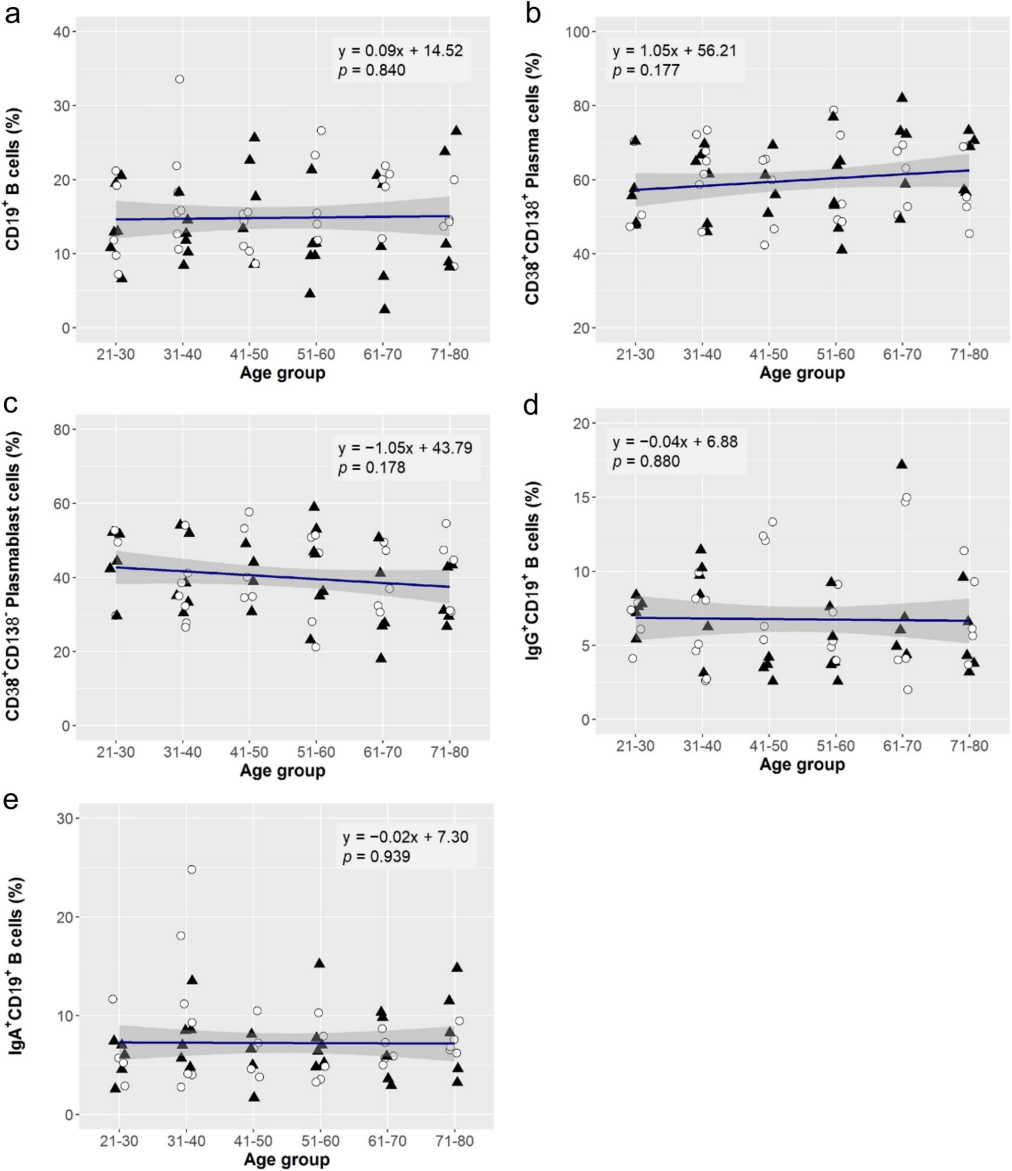
Age-associated changes in B cell subsets. Linear regression analysis to evaluate effect of aging on the frequencies of total B cells (**a**; CD19^+^ B cells), plasma cells (**b**; CD38^+^CD138^+^ Plasma cells), plasmablast cells (**c**; CD38^+^CD138^−^ Plasma cells), IgG^+^CD19^+^ B cells (**d**), and IgA^+^CD19^+^ B cells (**e**). The linear regression curve was plotted for the data of 67 subjects (33 females: open circle, 34 males: closed triangle), and its equation and *p*-value for the effect size of ageing are shown. The statistics of the analysis are summarised in Table S1.

With respect to the frequency of total B cells, plasma cells, plasmablast cells, IgG^+^CD19^+^ B cells, and IgA^+^CD19^+^ B cells, no significant age-associated change was observed in either of total subjects and the sex subgroups (Fig. 3 and Table S1).

### Effect of lifestyle factors on immune parameters

**Figure 4:**
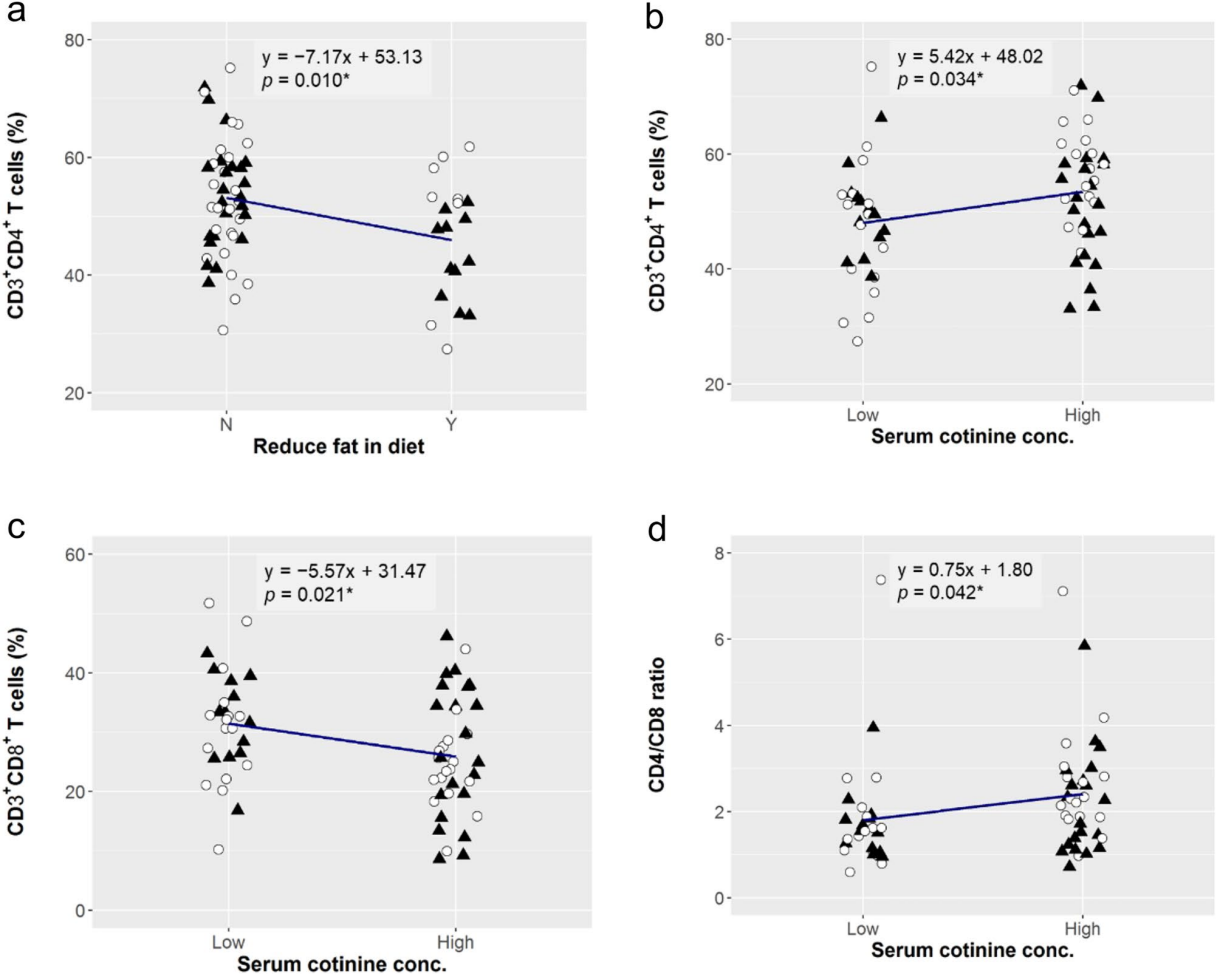
Effect of dietary fat intake and serum cotinine on CD4^+^ and CD8^+^ T cells. Linear regression analysis to evaluate the effect of reduce dietary fat intake on CD4 T cells (**a**), serum cotinine levels on CD4^+^ T cells (**b**), CD8^+^ T cells (**c**), and the ratio of CD4/CD8 T cells (**d**) in the PBMCs. The linear regression curve was plotted for the data of 67 subjects (33 females: open circle, 34 males: closed triangle), and its equation and *p*-value (*p < 0.050) for the effect size of the explanatory valuable are shown.

We observed a significant relationship only between fat intake and CD3^+^CD4^+^ T cells (estimate, − 7.17; *p* = 0.010; Fig. 4a), showing that individuals having a diet low in fat had significantly reduced frequency of CD3^+^CD4^+^ T cells in the PBMCs. The impact of smoking and chewing tobacco was also evaluated. We measured the metabolite of nicotine, cotinine levels in the serum of individuals, and the data of < 20 ng/ml was regarded as ‘low’ and that of ≥ 20 ng/ml was as ‘high’, respectively. Our results showed that high serum cotinine level is significantly associated with increased frequency of CD3^+^CD4^+^ T cells (estimate, 5.42; *p* = 0.034; Fig. 4b), reduced frequency of CD3^+^CD8^+^ T cells (estimate, − 5.57; *p* = 0.021; Fig. 4c), and higher CD4/CD8 ratio (estimate, 0.75; *p* = 0.042; Fig. 4d).

## Discussion

In this study, we assessed age-associated immune changes in the rural population in Maharashtra state of India. Several parameters of cellular immunity were found to be affected by ageing, especially in the frequency and activity of NK cell and T cell subpopulations (Figs. 1 and 2). We could not observe any significant age-associated changes in any parameters of humoral immunity (Fig. 3). The CD3^−^CD56^+^ NK cells represent 5–10% in the PBMCs^25^. Saxena et al.^20^ previously reported the value of the healthy Indian populations (*n* = 501) was 12.44 ± 7.55% (Mean ± SD) in the PBMCs, and our results were consistent with those observations (Table 1). We found an increased number but reduced functional capacity of NK cells with ageing (Fig. 1b,d). Similar results have been observed in the studies done in multiple countries where the number of NK cells significantly increased with age in the Spanish, French, and Australian populations, but not in the Austrian population^21,26^. There were few studies focusing on the functional capacities of NK cells, and Hazeldine et al. reported higher NK cell activity in the healthy young subjects (20–35 years) than the old ones (61–91 years)^27^. Our results (Fig. 1a,b) and others^28,29^ have shown that NK cell frequency increases from an early age in older individuals. However, the cytolytic and effector function of NK cells decreases with age. An increased number of cells may compensate for a decrease in the effector function of NK cells with age. However, a decrease in effector and cytolytic function of NK cells with age is due to the altered expression of several inhibitory and activating receptors on NK cells. The cumulative signalling threshold of these activating and inhibitory receptors dictates the function of NK cells^12,25^. It has been reported that activating receptors (NKp30 and NKp46) and one of the inhibitory receptors, CD94/NKG2A decreased in elderly individuals^27–29^. Other NK cell receptors are known as killer cell immunoglobulin-like receptors (KIRs) that have been known to have inhibitory as well as stimulatory functions on NK cells. Inhibitory KIRs (CD158a, CD158b, CD158e, and CD158i) were reported to significantly increase CD56^bright^ NK cells from an early age in elderly individuals^28,29^.

It is reported that older adults have an increased incidence of bacterial and fungal infections than younger individuals^30–32^. NK cells play an important role in fighting infections. A compromised NK cell cytotoxicity with age may hamper fighting the fungal infections^27,33,34^. In contrast, reduced IFN-γ secretion after activation with IL-2 represents immunosenescence with ageing^35^, which might affect the clearance of bacterial and viral infection in older individuals. Strategies that reduce, delay, or reverse the decline in NK cell function may have better implications for improving the health of older individuals.

Healthy lifestyles such as no smoking, physical exercise, and  daily intake of probiotics showed significantly higher NK cell activity^36,37^. These lifestyle factors plus geographical and genetic factors may play an important role in determining immunosenescence patterns between populations.

There was a significant increase in the proportion of CD4^+^ cells and a decrease in the proportion of CD8^+^ cells (Fig. 2a,b). Although we did not analyze the feature of decreased CD8^+^ T cells in the study, it has been reported that the decline in CD8^+^ T cells is more prominent in TCRαβ than in the TCRγδ subsets^26^. Previous studies have also shown a decrease in the percentage of naïve T cells and an increase in the percentage of effector memory T cells with ageing^38^. We also found a decreased number but augmented the functional ability of CD8^+^ cells (Fig. 2b,e). The age-associated changes in NK cells and T cells might be compensated by number and function to maintain homeostasis of the cellular immunity.

This study showed a significant age-associated CD4/CD8 ratio increase in a healthy Indian rural population (Fig. 2c). Normally, the ratio of CD4^+^/CD8^+^ in healthy adults is between 1.5 and 2.5; however, a wide heterogeny exists with CD4/CD8 ratio in different sex, ages, ethnicity, genetics, and exposure to various infections^39^. In adults, an altered ratio may indicate the disease activity in the body, such as immunodeficiency, autoimmunity, or viral such as HIV infection^39,40^. Hirokawa et al. also reported CD4/CD8 ratio showed a distinct age-related increase in the Japanese population^14^. These results indicate that the increase in CD4/CD8 ratio could be a potential marker of immunosenescence. It has been reported that T cell subsets such as CD4^+^ and CD8^+^ T cells show a wide range of frequency distribution in the different states of India at different geographical locations across the country^20^. The populations living in the Northern (Uttar Pradesh, Punjab, and Delhi) and Western (Maharashtra) states showed higher mean values of CD4/CD8 ratio (1.24 and above) as compared to the Southern states (0.91 in Tamil Nadu and 0.99 in Kerala). Another study in Rajasthan, a Northern state of India, supported this observation, reporting that CD4/CD8 ratio was 1.43 ± 0.56 in males and 1.78 ± 0.76 in females^41^. The study area was located in Maharashtra, the Western state of India, and the mean value of the CD4/CD8 ratio was higher than those in previous studies, indicating that the results might reflect the geographic or genetic variation in India.

Lifestyle variations between individuals have impacts on the immune system and immune defence mechanism. The two important concepts have been evolved through several epidemiological surveys: contributions of the environment (hygiene hypothesis) and of diet (dietary hypothesis) on the immune parameters of individuals. It has been shown that dietary salt intake has an impact on adaptive CD4^+^ T cells such as inflammatory Th17^42,43^, regulatory CD4^+^ T cells^44^, and innate immune cells, including macrophages^45,46^ and neutrophils^47^, and excessive salt intake is now recognized as a potential modulator of inflammatory and autoimmune diseases^48^. Dietary lipid administration also has been reported to modulate immune parameters^49^. Our results showed that the subgroup reducing fat in the diet showed a lower mean frequency of circulating CD4^+^ T cells in the PBMCs (Fig. 4a). Dietary fat intake is directly correlated with gut microbiota, and alteration in type and amount of the fat intake may lead to dysbiosis and inflammatory response^50–53^. Given that CD4^+^ T cells increase with ageing (Fig. 2a), excessive fat intake can be a factor in accelerating this age-associated change. Smoking and chewing tobacco alter the innate and adaptive immune systems and affect immune defence mechanisms^54,55^. High nicotine intake in the form of smoking or chewing tobacco is known to impact the development of several neurodegenerative diseases and cancers^55–57^. We observed that the level of serum cotinine, a metabolite of nicotine, related to the altered immune parameters with respect to CD4^+^ and CD8^+^ T cells (Fig. 2a,b), which was concordant with the previous results in the Indian population^20^. The role of CD8^+^ T cells has been well documented in infectious diseases, including viral infection and cancer^58^, and nicotine is known to ablate the function of CD8^+^ T cells to fight cancer^59^. Considering that CD8^+^ T cells are reduced with ageing (Fig. 2b), tobacco consumption can be regarded as a factor to the age-related change of CD8^+^ T cells.

Although there were some variations between the male and female subgroups in the effect size of ageing, the trends were in the same direction in all the immune parameters analyzed (Table S1). Steroid hormones affect both humoral and cellular immune responses, and estrogen is an important factor for different immunological responses between males and females^60^. In general, while estrogen action increases immune response^61^, it has been shown that mitochondrial oxidative stress is higher in male than in female and that higher levels of estrogens in females protects them against an age-related decline of the immune response by up-regulating the expression of antioxidants and longevity-related genes^62^. Yan et al. reported the ratio of activated B cells (CD20^+^CD69^+^ B cells) in the Australian populations significantly decreased with age only in the male subgroup but not in the female one^26^. Our study did not show any significant variations in the B cell subsets with age and sex (Fig. 3), and further investigations are necessary to obtain consistent results on sex differences in immune status.

The study had several limitations in its study design. We enrolled 67 subjects, but the sample size became much smaller when stratified according to their age (around 10 subjects in each age category). Another limitation is that the participants reside in a nearby area, and the results might contain regional characteristics of the study area. We used the qualitative questionnaire to collect the participants’ dietary information, but a quantitative assessment would be necessary to more specifically determine the effects of dietary factors. Furthermore, as this study was carried out in the Vadu HDSS area with a small sample size, this study's results could not be generalized to other parts of India. Further investigations involving a larger sample size across multiple regions would provide more concrete information on immunosenescence and related factors (sex and lifestyle) in the Indian population.

## Conclusions

The study provided evidence for immunosenescence in the Indian population. The cellular immunity was affected by ageing and some lifestyle factors of fat intake and tobacco consumption, while the humoral immunity was not significantly affected by either age, sex, and lifestyle factors. The frequency and number of NK cells increase but decrease its effector and cytolytic function with ageing. However, a detailed molecular mechanism of reduced NK cell cytotoxic activity with ageing need to be investigated further. Similarly, immunosenescence with ageing is a well-defined phenomenon in elder individuals. The current pandemic of COVID-19, where ageing is a co-morbidity factor in fighting the deadly infection, is due to compromised immunity. This also raises a concern that having an increased number of natural killer cells or cells with potent cytotoxic potential is an important parameter for better immunity to microbial infections. The strategies that promote the important immune parameter and reduce the immunosenescence will help to give better immunity to infections in elderly populations.

## Supplementary Information

Supplementary Figure S1.

Supplementary Tables.

## Data Availability

All data generated or analysed during this study are included in this published article.
