# Supplementary material for: Changes in immunological parameters by ageing in rural healthy Indian adults and their associations with sex and lifestyle
Source: Sci Rep. 2022 Sep 2;12:15012. doi: 10.1038/s41598-022-19227-z (PMC9438881; doi:10.1038/s41598-022-19227-z)
Supplement: Supplementary file 1 — Supplementary Figure S1. [file 41598_2022_19227_MOESM1_ESM.docx]

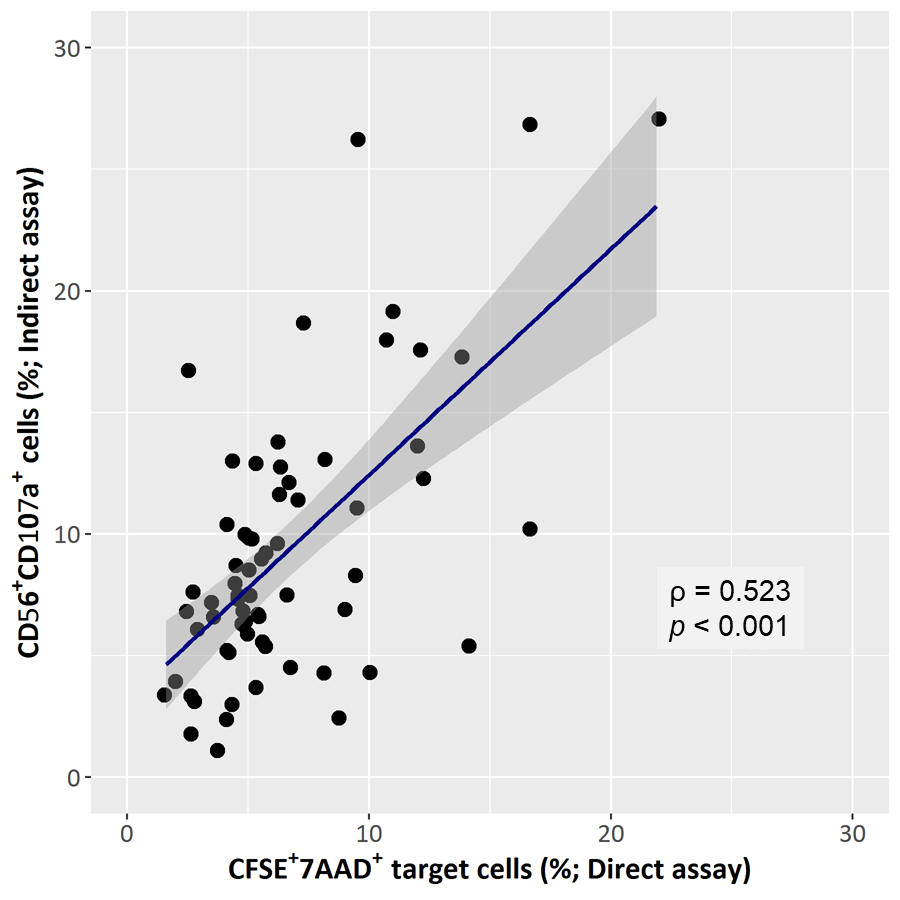


**Figure S1.** Relationship of the results between assays to evaluate NK cell activity. Correlation of 67 blood samples between the direct assay (CFSE-based NK cell cytotoxic activity) and indirect assay (degranulation potential of NK cells). Linear regression curve with 95% CI was plotted, and Spearman’s Rank correlation coefficient (ρ) and *p*-value were calculated.
