# Supplementary material for: Changes in immunological parameters by ageing in rural healthy Indian adults and their associations with sex and lifestyle
Source: Sci Rep. 2022 Sep 2;12:15012. doi: 10.1038/s41598-022-19227-z (PMC9438881; doi:10.1038/s41598-022-19227-z)
Supplement: Supplementary file 2 — Supplementary Tables. [file 41598_2022_19227_MOESM2_ESM.docx]

**Table S1.** Statistics of generalized linear regression analysis predicting effect of aging on immunological parameters.

| **Item** | **Total (*n* = 67)** | | |  | **Female (*n* = 33)** | | |  | **Male (*n* = 34)** | | |
| --- | --- | --- | --- | --- | --- | --- | --- | --- | --- | --- | --- |
|  | **Estimate** | **95% CI** | ***p*-value** |  | **Estimate** | **95% CI** | ***p*-value** |  | **Estimate** | **95% CI** | *p*-value |
| CD3^−^CD56^+^ NK cells (%) | 1.86 | [0.85, 2.87] | < 0.001** |  | 0.94 | [−0.52, 2.40] | 0.215 |  | 2.73 | [1.37, 4.10] | < 0.001** |
| CD3^−^CD56^+^ NK cells (counts/10^5^ PBMCs) | 972.8 | [355.5, 1590.1] | 0.003** |  | 652.5 | [−293.9, 1598.9] | 0.186 |  | 1279.4 | [467.1, 2091.8] | 0.004** |
| CFSE^+^7AAD^+^ target cells (%) | −0.18 | [−0.75, 0.38] | 0.530 |  | −0.22 | [−1.14, 0.70] | 0.646 |  | −0.13 | [−0.78, 0.52] | 0.697 |
| CD107a^+^CD56^+^ NK cells (%) | −0.88 | [−1.68, −0.08] | 0.034* |  | −1.05 | [−2.13, 0.03] | 0.065 |  | −0.72 | [−1.91, 0.48] | 0.247 |
| CD3^+^CD4^+^ T cells (%) | 2.27 | [0.87, 3.66] | 0.002** |  | 3.18 | [1.10, 5.26] | 0.005** |  | 1.40 | [−0.46, 3.26] | 0.149 |
| CD3^+^CD8^+^ T cells (%) | −3.04 | [−4.25, −1.83] | < 0.001** |  | −2.49 | [−4.25, −0.73] | 0.009** |  | −3.57 | [−5.25, −1.88] | < 0.001** |
| CD4/CD8 ratio | 0.43 | [0.24, 0.62] | < 0.001** |  | 0.45 | [0.18, 0.72] | 0.003** |  | 0.42 | [0.15, 0.69] | 0.005** |
| IFN-γ^+^CD4^+^ T cells (%) | 0.07 | [−0.74, 0.88] | 0.870 |  | −0.17 | [−1.35, 1.00] | 0.777 |  | 0.30 | [−0.84, 1.44] | 0.613 |
| IFN-γ^+^CD8^+^ T cells (%) | 5.05 | [3.08, 7.02] | < 0.001** |  | 5.90 | [3.18, 8.62] | < 0.001** |  | 4.24 | [1.35, 7.13] | 0.007** |
| CD19^+^ B cells (%) | 0.09 | [−0.78, 0.96] | 0.840 |  | 0.17 | [−1.02, 1.36] | 0.778 |  | 0.02 | [−1.23, 1.27] | 0.977 |
| CD38^+^CD138^+^ Plasma cells (%) | 1.05 | [−0.46, 2.57] | 0.177 |  | 0.21 | [−2.01, 2.43] | 0.854 |  | 1.85 | [−0.23, 3.93] | 0.091 |
| CD38^+^CD138^−^ Plasmablast cells (%) | −1.05 | [−2.56, 0.46] | 0.178 |  | −0.21 | [−2.43, 2.01] | 0.855 |  | −1.85 | [−3.93, 0.23] | 0.091 |
| IgG^+^CD19^+^ B cells (%) | −0.04 | [−0.54, 0.47] | 0.880 |  | 0.22 | [−0.55, 0.99] | 0.582 |  | −0.28 | [−0.94, 0.37] | 0.404 |
| IgA^+^CD19^+^ B cells (%) | −0.02 | [−0.61, 0.57] | 0.939 |  | −0.38 | [−1.35, 0.59] | 0.451 |  | 0.32 | [−0.37, 1.01] | 0.375 |

The effect size of aging (6 age groups: 21–30, 31–40, 41–50, 51–60, 61–70, and 71–80 years) on each immunological parameter was evaluated by linear regression model (* *p* < 0.050, ** *p* < 0.010).

**Table S2.** Statistics of normality and homoscedasticity tests in the generalized linear regression.

| **Item** | **Normality** | |  | **Homoscedasticity** | |
| --- | --- | --- | --- | --- | --- |
|  | **W** | ***p*-value** |  | **GQ** | ***p*-value** |
| CD3^−^CD56^+^ NK cells (%) | 0.956 | 0.018* |  | 0.996 | 0.505 |
| CD3^−^CD56^+^ NK cells (counts/10^5^ PBMCs) | 0.924 | < 0.001* |  | 0.982 | 0.521 |
| CFSE^+^7AAD^+^ target cells (%) | 0.865 | < 0.001* |  | 0.555 | 0.947 |
| CD107a^+^CD56^+^ NK cells (%) | 0.917 | < 0.001* |  | 0.288 | 1.000 |
| CD3^+^CD4^+^ T cells (%) | 0.966 | 0.062 |  | 0.800 | 0.733 |
| CD3^+^CD8^+^ T cells (%) | 0.947 | 0.007* |  | 1.159 | 0.342 |
| CD4/CD8 ratio | 0.777 | < 0.001* |  | 1.704 | 0.783 |
| IFN-γ^+^CD4^+^ T cells (%) | 0.902 | 0.949 |  | 0.755 | 0.077 |
| IFN-γ^+^CD8^+^ T cells (%) | 0.992 | < 0.001* |  | 1.676 | 0.071 |
| CD19^+^ B cells (%) | 0.968 | 0.085 |  | 1.112 | 0.384 |
| CD38^+^CD138^+^ Plasma cells (%) | 0.958 | 0.031* |  | 1.329 | 0.223 |
| CD38^+^CD138^−^ Plasmablast cells (%) | 0.958 | 0.031* |  | 1.329 | 0.223 |
| IgG^+^CD19^+^ B cells (%) | 0.920 | < 0.001* |  | 0.739 | 0.793 |
| IgA^+^CD19^+^ B cells (%) | 0.851 | < 0.001* |  | 1.659 | 0.088 |

The assumptions of normality and homoscedasticity in the generalized linear regression (Table S1) were tested by the Shapiro-Wilk test and the Goldfeld-Quandt test, respectively (* *p* < 0.050).

**Table S3.** Statistics of non-parametric linear regression analysis predicting effect of aging on immunological parameters.

| **Item** | **Total (*n* = 67)** | | |
| --- | --- | --- | --- |
|  | **Estimate** | **95% CI** | ***p*-value** |
| CD3^−^CD56^+^ NK cells (%) | 1.93 | [1.51, 2.27] | < 0.001** |
| CD3^−^CD56^+^ NK cells (counts/10^5^ PBMCs) | 705.00 | [534.7, 1053.8] | < 0.001** |
| CFSE^+^7AAD^+^ target cells (%) | 0.03 | [−0.28, 0.13] | 0.671 |
| CD107a^+^CD56^+^ NK cells (%) | −0.30 | [−0.84, −0.10] | 0.011* |
| CD3^+^CD4^+^ T cells (%) | 3.26 | [2.17, 3.48] | < 0.001** |
| CD3^+^CD8^+^ T cells (%) | −3.78 | [−4.22, −3.28] | < 0.001** |
| CD4/CD8 ratio | 0.39 | [0.32, 0.43] | 0.703 |
| IFN-γ^+^CD4^+^ T cells (%) | −0.03 | [−0.45, 0.28] | < 0.001** |
| IFN-γ^+^CD8^+^ T cells (%) | 5.65 | [4.12, 6.12] | < 0.001** |
| CD19^+^ B cells (%) | 0.18 | [−0.29, 0.51] | 0.673 |
| CD38^+^CD138^+^ Plasma cells (%) | 0.90 | [0.34, 1.80] | 0.004** |
| CD38^+^CD138^−^ Plasmablast cells (%) | −0.90 | [−1.84, −0.34] | 0.004** |
| IgG^+^CD19^+^ B cells (%) | −0.43 | [−0.48, −0.09] | 0.006** |
| IgA^+^CD19^+^ B cells (%) | 0.10 | [−0.09, 0.34] | 0.218 |

The effect size of aging (6 age groups: 21–30, 31–40, 41–50, 51–60, 61–70, and 71–80 years) on each immunological parameter was evaluated by non-parametric linear regression model (* *p* < 0.050, ** *p* < 0.010).
